# An optimized procedure for the design and evaluation of Ecotilling assays

**DOI:** 10.1186/1471-2164-9-510

**Published:** 2008-10-30

**Authors:** Stefan Coassin, Anita Brandstätter, Florian Kronenberg

**Affiliations:** 1 Division of Genetic Epidemiology; Department of Medical Genetics, Molecular and Clinical Pharmacology; Innsbruck Medical University, Innsbruck, Austria

## Abstract

**Background:**

Single nucleotide polymorphisms (SNPs) are the most common form of genetic variability in the human genome and play a prominent role in the heritability of phenotypes. Especially rare alleles with frequencies less than 5% may exhibit a particularly strong influence on the development of complex diseases. The detection of rare alleles by standard DNA sequencing is time-consuming and cost-intensive. Here we discuss an alternative approach for a high throughput detection of rare mutations in large population samples using Ecotilling embedded in a collection of bioinformatic analysis tools. Ecotilling originally was introduced as TILLING for the screening for rare chemically induced mutations in plants and later adopted for human samples, showing an outstanding suitability for the detection of rare alleles in humans. An actual problem in the use of Ecotilling for large mutation screening projects in humans without bioinformatic support is represented by the lack of solutions to quickly yet comprehensively evaluate each newly found variation and place it into the correct genomic context.

**Results:**

We present an optimized strategy for the design, evaluation and interpretation of Ecotilling results by integrating several mostly freely available bioinformatic tools. A major focus of our investigations was the evaluation and meaningful economical combination of these software tools for the inference of different possible regulatory functions for each newly detected mutation.

**Conclusion:**

Our streamlined procedure significantly facilitates the experimental design and evaluation of Ecotilling assays and strongly improves the decision process on prioritizing the newly found SNPs for further downstream analysis.

## Background

Several studies have shown that especially rare alleles with frequencies below 5% (referred to as "rare" alleles) may have a strong impact on quantitative traits and complex diseases [1-6]. Furthermore, recent genome-wide association studies on qualitative and quantitative traits highlighted the importance of genetic variation outside coding regions or even gene regions [7-11] which sheds new light on these in the past often neglected intronic and even intergenic regions, which harbour nearly 90% of all SNPs [12].

The discovery of rare alleles in large populations is challenging. Standard DNA sequencing of sizeable gene regions in large study populations with the aim to identify the contribution of rare mutation is time-consuming and cost-intensive. Depending on the sequencing quality, heterozygous mutations may be lost in background noise. Thus, Sanger sequencing shows limitations for the discovery of rare and hence mostly heterozygous mutations [13,14]. In addition, many of the current pre-screening technologies such as single strand conformational polymorphism analysis or gradient gel electrophoresis are laborious, target only relatively small portions of DNA (as it is the case for dHPLC) or are not capable for high throughput. Techniques that allow an inexpensive but high throughput detection of rare mutations with high sensitivity are thus of high interest [13,15].

A technology that promises a cost-effective screening of 15–20 kb large gene regions for rare mutations with high sensitivity is Ecotilling. Ecotilling was originally developed as TILLING for large scale screenings of chemically induced mutations in plants [16], and later adopted for human samples [13]. An important advantage of this technology is its capability to detect mutations in assays with up to eightfold pooled DNA samples (which corresponds in case of a heterozygous mutation to the detection of one mutation in out of 16 strands), thus boosting the throughput, lowering the costs and enabling the detection of multiple homozygous mutations [13]. Since up to two runs are possible in a typical working day, the investigation of up to nearly 2.3 Mb (192 samples, 1500 bp each sample) in a single day becomes possible even in a relatively small research laboratory.

Despite its obvious benefits the use of Ecotilling for research projects in human medical research is still limited. A possible reason for this may be the complex process of translating the image information to sequence annotation, the interpretation of the findings and the selection process of newly discovered SNPs for further downstream analysis. For example, a crucial point for the interpretation and evaluation of the gel data is the knowledge about the location of the detected mutations within the gene. Especially when analyzing a SNP-rich organism like homo sapiens, a fast way to localize the signals relative to the analyzed PCR fragment and relative to already known polymorphisms or known functional elements is essential for a fast and efficient examination of Ecotilling gels. While large TILLING facilities apply bioinformatic tools to map and store the mutations found by TILLING (e.g. as done at the Seattle TILLING Project [17]), no suitable software solution exists for relatively small laboratories.

Unlike DNA sequencing, Ecotilling data have to be manually translated from a "picture form" to a more accessible "DNA sequence form". The efficient realization of this step poses an essential problem in the evaluation of Ecotilling gels. Here we present an optimized strategy for the design, analysis and interpretation of human Ecotilling experiments. Our proposed procedure involves an appropriate usage of mostly freely available software tools and enables a straight-forward post-laboratory analysis of Ecotilling results without the need of sophisticated laboratory information management solutions.

## Methods

### Ecotilling

The principles of Ecotilling are described in detail in references [13,16] and [18]. Briefly, the basic step of TILLING is the amplification of a region of interest of up to 1.5 kb using two primers labeled with different infrared fluorochromes, followed by heat-denaturation and slow cooling to allow the formation of heteroduplexes in the presence of heterozygous mutations. These are digested with Cel-I, a mismatch-specific endonuclease which cuts at the 3' end of mismatches [19]. The digestion products are size-fractionized on a LI-COR slab gel sequencer (DNA Analyzer 4300S; LI-COR Biosciences, Lincoln, NE, USA). The simultaneous detection at both fluorescent dye wavelengths yields two gel-images for every analyzed fragment, with both images showing the undigested full size-product (which is still bearing both 5' dyes). In the case that a digested mutation is present, each detection channel shows an additional signal where the sum of the size of both fragments is equal to the length of the full size product. Since the reaction conditions are supposed to avoid a full digestion of the PCR products (and therefore not every mismatch is recognized in every strand), multiple mutations per fragment and their approximate position (± 10 bp) can be detected. Furthermore, since every real mutation should produce complementary bands in the two detection channels, an immediate quality control of each signal is possible [18].

### Design of Ecotilling experiments

The design of Ecotilling is done by integrating the functions of Vector NTI (Advance 10; Invitrogen Corporation, Carlsbad, CA, USA; ), the Ensembl Database , the Genomatix Suite (Genomatix Software GmbH, Munich, Germany; ) and some software for primer design such as VisualOMP (DNA Software, Ann Arbor, MI, USA) or Primer3 .

### Data analysis of Ecotilling experiments

The data analysis procedure includes three software tools: Vector NTI, GelBuddy ([20]; ) and sequencing analysis software solutions such as SeqScape 2.6 (Applied Biosystems, Foster City, CA, USA) or Sequencher 4.8 (GeneCodes, Ann Arbor, MI, USA).

### Interpretation of Ecotilling experiments

The interpretation of Ecotilling gels is based on the gene model created in Vector NTI and the findings can further be investigated using different bioinformatic tools, depending on the identity, type and location of each mutation.

Coding SNPs are evaluated using a Vector NTI data set containing the respective cDNA and the homology-based web tools Polyphen  and SIFT .

The tools for investigation of possible functional effects of SNPs in non-coding regions comprehend the Genomatix Software Suite for investigation of transcription factor binding sites, TARGETSCAN ([21]; ), PATROCLES ([22]; ) and polymiRTS ([23]; ) for miRNA evaluation, the VISTA Genome Browser  for long range alignments and the UCSC Browser  for evaluating unknown regulatory regions through analyzing conservational pattern and ESPERR scores [24].

## Results and Discussion

**Figure 1:**
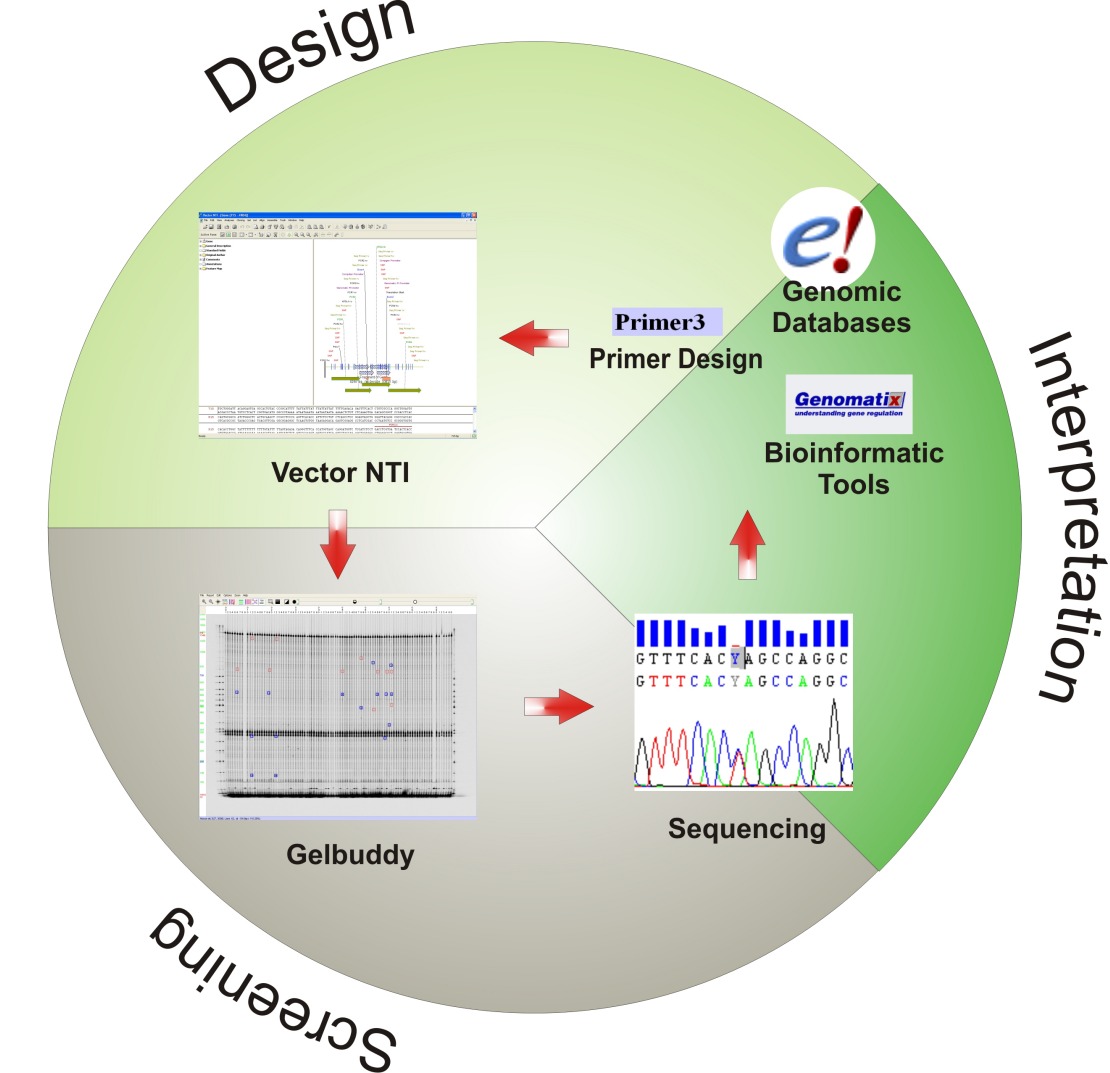
**Overview of the proposed workflow**. The process starts in the upper right corner. *Design: *Information from several sources is integrated into the vector NTI data set, which then superimposes all relevant information on the gene's sequence and includes all information regarding positions of primers and PCR fragments. *Screening: *Every signal derived from the Ecotilling screening is checked in Vector NTI for already known features at that position. The sequencing primers can easily be chosen for confirmation of so far unknown signals. *Interpretation: *Once the exact identity of a signal is known, the variation is checked for potential functional relevance using appropriate bioinformatic tools. Any new variation is annotated in Vector NTI and therefore directly taken into account when analyzing the next Ecotilling run.

The screening of whole genetic regions in large populations has to be done in batches, by splitting samples into multiples of 96 depending on the pooling strategy (e.g. 384 sample batches for a pooling of 4 samples per lane) and dividing gene regions into overlapping fragments of a maximum size of 1.5 kb. In order to efficiently choose the samples that may be subjected to confirmatory sequencing, it is crucial to keep a general overview of the results during the entire process. The use of Vector NTI as an underlying sequence database supports the design and evaluation of Ecotilling experiments by efficiently placing new mutations in relation to already known structures and functional predictions. At any time new results can be integrated into all subsequent analyses. Figure 1 gives an overview of our approach. The two movies online available are highly illustrative to follow the proposed work flow described in detail below.

### 1. Design of Ecotilling experiments

#### Proposed workflow

##### Construction of the Vector NTI design overview

**Figure 2:**
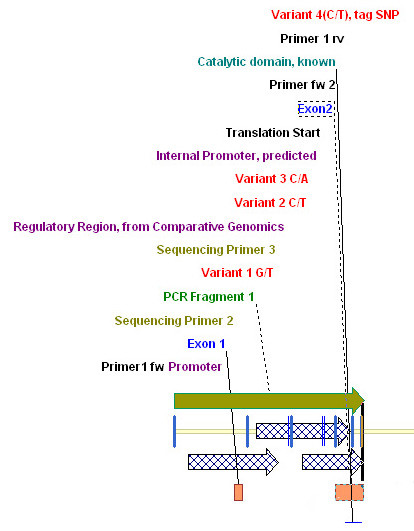
**Screenshot of Vector NTI showing an example of a data set prepared for Ecotilling**. The Vector NTI interface is divided in 3 panels: a graphical overview, an annotated sequence and a textual overview. The graphical overview displays several features, including known SNPs (red), regulatory regions (blue shaded arrows), exons (orange boxes and blue annotation), known functional features, as catalytic domains (cyan), sequencing primers (olive-colored), amplification primers (black) and the location of PCR fragments (olive colored bars and green annotation). The distance between any of the genetic elements can be easily determined by dragging the mouse over the sequence strand (see also Figure 3 and additional file 3). This feature is particularly important for Ecotilling, which is in principle based on differing electrophoretic motilities of the digestion products of different lengths. This figure shows a section of the graphical overview of the whole data set. For an entire screenshot of how Vector NTI is presenting the data, please see additional file 2.

First of all, an overview of all relevant information about the investigated genetic region is constructed in Vector NTI. To this end, the genetic target region including all variation features is exported from Ensembl as a GenBank file using the "Export from region" function with subsequent import into Vector NTI (see additional file 1). This provides a sequence with already all known mutations annotated as basis for all subsequent annotations. This is routinely done as the first step for each new experiment, followed by the annotation of the exons and retrieval of all known or predicted promoter regions (including alternative promoters) derived from the Genomatix Suite as well as annotation of all other relevant information such as the location of known functional genetic elements or bioinformatic predictions (Figure 2 and additional files 1 and 2). These steps can be easily done in less than one hour.

Finally, the construction of a second parallel Vector NTI project using only the cDNA can be highly beneficial for a fast evaluation of the effects of exonic SNPs, since Vector NTI allows easy one-click translation of coding sequences.

##### Primer design

The target region is selected in the Vector NTI interface and pasted into any primer design program. The design of the amplification primers yielding overlapping PCR fragments is crucial to fully cover each gene region, since Ecotilling shows a lower resolution in the first approximately 150 bp of each fragment. After design, the primer binding sites can easily be checked in Vector NTI to ensure the optimal position of each primer regarding to any regions of particular interest.

The same procedure is followed for the sequencing primers. By designing and optimizing them immediately together with the PCR fragments, it is possible to set up a high throughput mutation validation routine for the newly detected mutations of each target gene, where any detected mutations can be readily sequenced. This allows sequencing of each detected mutation within 48 hours from the Ecotilling run.

#### Comments

**Figure 3:**
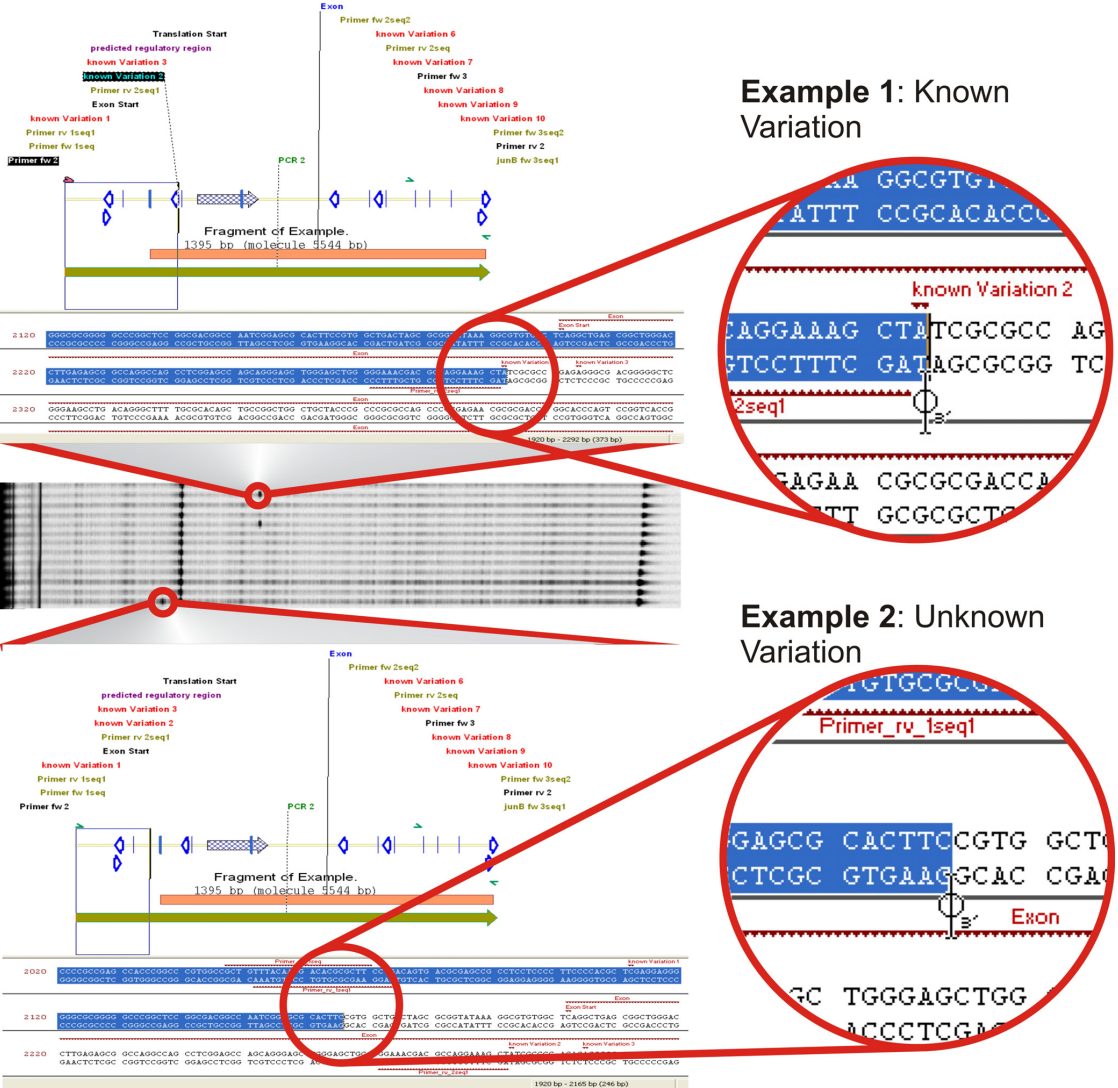
**Demonstration of the evaluation of Ecotilling signals using Vector NTI**. This image shows a section of an Ecotilling gel targeting the 5' gene region of *JUNB *and showing two different signals. Each lane contains pooled PCR fragments of four different individuals. The DNA extracts were normalized and pooled prior to PCR amplification. *Upper panel: *A signal appears in two lanes at approximately 370 bp from the forward primer. The measured distances are reported in the lower right corner of the Vector NTI window. By looking up the respective position in Vector NTI shows that two adjacent SNPs are known at that position (consider the zoomed sequence) and a potentially regulatory region (shaded arrow) is located nearby downstream. *Lower panel: *A signal is located at approximately 245 bp from the forward primer. Inspection of the respective region in the Vector NTI data set reveals that no so-far known SNP is located at the respective region (considering that no SNP is annotated to the sequence). The signal is located immediately upstream of the exon (orange bar) and near a potentially regulatory region (shaded arrow and purple annotation). A reverse sequencing primer is located nearby downstream (bold blue arrow and green annotations). This procedure is shown in more detail by the additional file 3 (movie-2.avi).

The use of Vector NTI for the design of Ecotilling experiments has several benefits. Due to the high density of known SNPs in the human genome, a crucial point for an easy evaluation of Ecotilling runs is the ability to readily discriminate between known and unknown mutations (see Figure 3 and additional file 3) and to differentiate true-positive signals from signals produced by other genetic structures such as poly-T stretches. Since by-hand annotation of all known variations over large genetic regions is extremely time-consuming, the possibility of Vector NTI to import this information directly from Ensembl.org is highly beneficial.

The resulting data set represents the basis for the subsequent primer design. Optimal primer design is essential for the entire experiment as primers are often designed without exact knowledge of the location of potentially interesting regions, resulting sometimes in several PCR redesigns. While the position of coding regions is usually known and taken into account, potential regulatory regions are seldom precisely considered during primer design, although they harbor the majority of (e)QTL loci [25]. This happens mostly because information about their location is hardly to retrieve in a well arranged form. While the use of many of the publicly available databases is often not really intuitive, we found that the Genomatix Database represents a powerful and user-friendly, although proprietary source of information on regulatory elements. Therefore the integration of information about regulatory regions into the Vector NTI seems a strong advantage for mutation screening in epidemiological studies, especially given the recent results outside of coding regions or even gene regions as discussed above [7-11]. Figure 2 and additional file 2 provide an example for a complete Vector NTI data set for Ecotilling screenings.

### 2. Data analysis of Ecotilling experiments

#### Proposed workflow

##### Ecotilling image analysis

The data analysis starts with the analysis of the Ecotilling gel images using GelBuddy [20]. At the same time, the corresponding Vector NTI project is opened and by blinding out the remaining molecule, only the current PCR product remains displayed. When a differential signal is detected in GelBuddy, its position can easily be mapped in Vector NTI by dragging the mouse from the end of the PCR-product corresponding to the analyzed detection channel across the PCR product and progressively marking the product (Figure 3). Vector NTI automatically counts the marked bases, giving a convenient way to determine the sequence context of each signal. This allows seeing if the signal corresponds to a known SNP or to another structure producing positive Ecotilling signals, such as poly-T regions. At the same time, the region in which the signal is located can be checked for any functionally interesting features and thus, the possible importance of any new signal can rapidly be assessed. The visual determination of the position and context of each new mutation strongly facilitates the choice of sequencing primers for the validation of the mutation. The additional file 3 (movie-2.avi, available online) exemplifies this procedure.

##### Sequencing analysis

Since the exact position by Ecotilling can only be determined with an accuracy of ± 5–10 bp, prioritized signals should be sequenced to determine their exact identity. Any new mutation is then directly annotated in Vector NTI and considered in any subsequent gel run.

#### Comments

An important point that distinguishes human Ecotilling from Ecotilling or TILLING in all other model organisms is the high number of already known SNPs. This makes the screening for new and rare polymorphisms in humans more challenging and strongly requires an exact knowledge of all already known polymorphisms in the analyzed region.

The described gel evaluation procedure gives a fast and straight-forward way to evaluate any signal on the gel. The rapid determination of the position of a signal and easy inspection of its sequence context for any functional relevance, therefore, simplifies the analysis of Ecotilling images and the choice of the right sequencing primers. This allows creating a high-throughput screening *and *sequencing procedure.

The definitive identification of previously unknown, possibly heterozygous mutations by sequencing is often difficult, especially in sequence contexts, where the required high quality reads may be hard to obtain. A crucial point when choosing the appropriate sequence analysis software is the ease to compare multiple electropherograms, as offered by SeqScape or Sequencher. Especially the latter offers multiple tools for in-depth analysis of electropherograms and is in our eyes especially appropriate for the confirmation of insertion/deletion polymorphisms, which are otherwise quite difficult to ascertain.

### 3. Interpretation of Ecotilling experiments

#### Proposed workflow

The interpretation of Ecotilling results starts by inspecting the position of new mutations in Vector NTI for already known or in-silico predicted genetic elements. Exonic SNPs are annotated using the cDNA data set and their effect on the protein sequence is determined. The possibility to easily translate DNA sequences into amino acid sequence in Vector NTI enables a fast evaluation of the effects of SNPs on the amino acid composition and their position relative to known protein domains. The effect of a non-synonymous SNP are finally evaluated using the freely available online tools Polyphen and SIFT.

The evaluation of mutations in non-coding regions is more challenging due to the general lack of knowledge about cis-regulatory elements outside the restricted core promoter [26] and in-depth analyses require strong bioinformatic support.

The effect of sequence variations on putative transcription factor binding sites can be assessed using SNPInspector, MatInspector [27], ModelInspector [28] and other tools from the Genomatix' GEMS Launcher.

Possible effects on miRNA binding sites can be estimated with the web-tools TARGETSCAN [21], PATROCLES [22] and polymiRTS [23].

The potential functional relevance of the region surrounding an interesting SNP can be evaluated by analyzing the evolutionary conservational pattern and potential regulatory functions in the UCSC genome browser. Potential regulatory elements can be found with the ESPERR Regulatory Potential, which is highly accurate in distinguishing regulatory from neutral DNA stretches [24] and by investigating the nucleotide conservation with the UCSC and VISTA Genome Browsers. A typical example for the application of these methods to investigate the influence of genetic variability on phenotypes of interest can be found in our recent genome-wide association study on HDL cholesterol (reference [7] including the respective Supplementary Material).

#### Comments

A large amount of information on the functionality of different human genetic regions (regulatory regions, promoters, catalytic domains, etc.) can be obtained through the literature and databases. In principle, all available information has to be taken into consideration for the interpretation and evaluation of newly discovered mutations. Thus, a quick but thorough interpretation of Ecotilling results is the most challenging step in the post-laboratory analysis.

While the evaluation of coding SNPs is usually straightforward, the evaluation of SNPs outside of coding regions is more difficult. We propose here an interpretation procedure dedicated to the analysis of functional effects of SNPs derived from genetic epidemiological studies, which aims to consider as many different regulatory layers as possible.

Although several powerful databases for transcription factor binding sites and mammalian promoters were made available in the last years (such as JASPAR [29] and TRANSFAC [30] or MPromDB [31] and EPD [32]), these are often too laborious for a quick evaluation and lack the integration of different layers of information.

An optimal tool collection for this purpose is represented by the Genomatix Software Suite, which includes several tools ranging from literature mining tools (Bibliosphere, LitInspector) to an annotated genome browser (Eldorado) and tools for searching and evaluating transcription factor binding sites (MatInspector [27], SNPInspector). The integration of all tools into one common HTML-interface helps to consider several different layers of functional information, facilitates the prioritization of mutations for downstream analysis and enables the evaluation of potential effects of new SNPs without strong bioinformatic expert knowledge.

As transcription factor binding sites can be located elsewhere than upstream of a gene [33], we emphasize that all SNPs and their surrounding sequence should routinely be investigated for transcription factor binding sites.

Recently it was shown that SNPs within 3'-UTRs can affect gene expression by affecting miRNA binding sites [22,34]. It was suggested that up to one third of all human genes may be targeted by miRNAs [35,36], indicating that SNPs affecting miRNA binding sites may be a common phenomenon. Therefore SNPs in 3' UTR regions should always be evaluated for affecting miRNA binding sites, and intergenic SNPs should be checked for effects on any known miRNAs (see Methods section).

## Conclusion

Recent advances in sequencing technologies promise to revolutionize genetics by enabling genome-wide and highly multiplexed mutation discovery studies [37]. These technologies are still challenging and demand both considerable wet lab capacities and extensive bioinformatic support. For the time being, next generation sequencing is not adapted to rapidly screen one region in many different samples. We found that in this case, Ecotilling shows outstanding performance for cost-effective screening of single gene regions in large sample numbers and is therefore especially appropriate for mutation screening studies that may not reach the target region size for next-generation sequencing. Recent technological advances did further improve the performance of Ecotilling by allowing the use of capillary electrophoresis systems [38]. This brings several benefits such as the use of even higher samples pools, reduced background signal and higher throughput due to automated sample loading [38,39].

**Figure 4:**
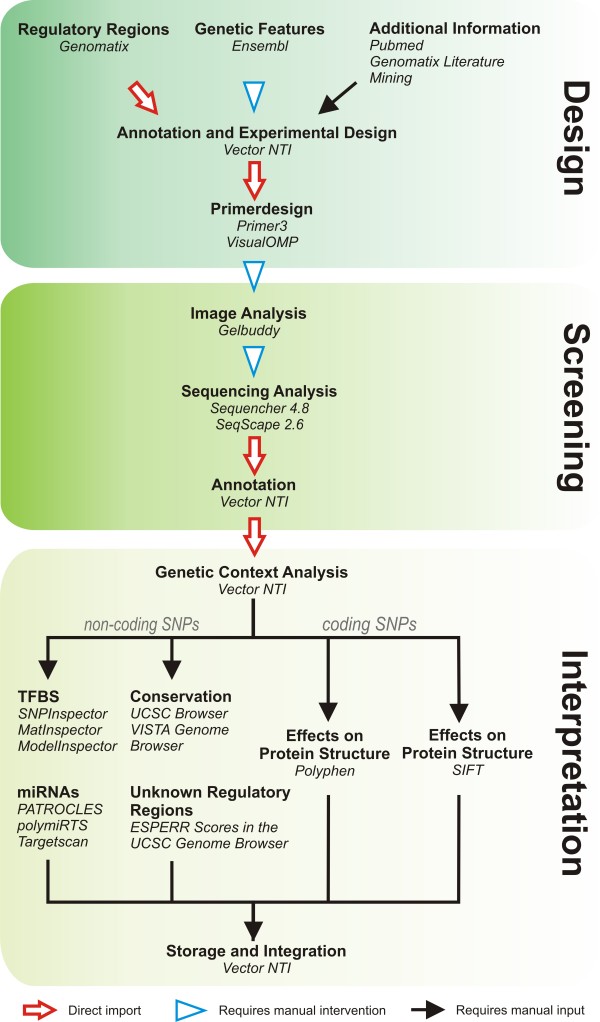
**Quick reference guide giving an overview of the workflow including the tools used for each step**. The arrow types show if the respective step can be done simply by importing a data file or by "copy and paste" procedures or if a more or less extended manual intervention is needed. Note: the extent of manual intervention in importing the Ensembl data set depends on the number of genetic structures that are imported. If only SNP data and exon location are imported, this can be done by importing the SNP data as GenBank file and copy-pasting the exons.

The major problem with the implementation of Ecotilling as a screening tool in small research laboratories is the lack of an integrated software solution for the design, evaluation and interpretation of Ecotilling assays and results. In the course of establishing several Ecotilling projects, screening up to 12 kb in more than to 1500 samples, we developed a streamlined procedure for setting up the experiment, dealing with the results and interpret the possible functions of newly discovered SNPs widely by applying freely available software tools (figure 4).

Although we did not develop a completely new software solution, we describe in detail an optimized procedure for a thorough evaluation of Ecotilling results, which summarizes know-how on a widespread variety of freely available bioinformatic tools. The basic problem to translate findings from mutation screenings into a more accessible sequence database and evaluate them in a quick yet comprehensive manner, is not restricted to screenings employing Ecotilling. This may apply also to screening projects employing other technologies. Especially with the advent of next-generation sequencing technologies and the resulting huge amount of data that any geneticist will face, our approach may help to keep an overview in every evaluation process involving different layers of information. Thus, we believe that our approach may be interesting also for laboratories using any other mutation screening technology.

## Abbreviations

(e)QTL: (expression) Quantitative Trait Locus; CAGE: Cap Analysis Gene Expression; dHPLC: denaturing High Performance Liquid Chromatography; ESPERR: Evolutionary and Sequence Pattern Extraction through Reduced Representations; TILLING: Targeting Induced Local Lesions IN Genomes; UTR: untranslated region.

## Authors' contributions

SC established Ecotilling in our laboratory and assembled the effective workflow of the described bioinformatic tools. SC and AB prepared the first draft of the manuscript and AB contributed several ideas to this workflow. FK prepared the introduction of Ecotilling in our lab, supervised the project and contributed to the discussion and writing of the manuscript.

## Supplementary Material

Additional file 1 **Using the gene *MYC *we show the first steps of the data set creation.** This includes the retrieval of a genomic gene sequence in the Ensembl data base and its subsequent import into Vector NTI including the location of known variations. Finally, an example for a fully annotated Vector NTI data set is showed. Click here for file

Additional file 2 **This image shows a screenshot of a full Vector NTI data set prepared for Ecotilling, as described in figure legend 2**. Figure 2 shows a section of this image. Click here for file

Additional file 3 **This file shows the integrated use of GelBuddy and Vector NTI to interpret signals on an Ecotilling Gel and choose the appropriated sequencing primers for sequencing confirmation.** The example uses genomic information of *JUNB *as shown in Figure 3. Click here for file
