# Supplementary figures and images for: An optimized procedure for the design and evaluation of Ecotilling assays
Source: BMC Genomics. 2008 Oct 30;9:510. doi: 10.1186/1471-2164-9-510 (PMC2586031; doi:10.1186/1471-2164-9-510)

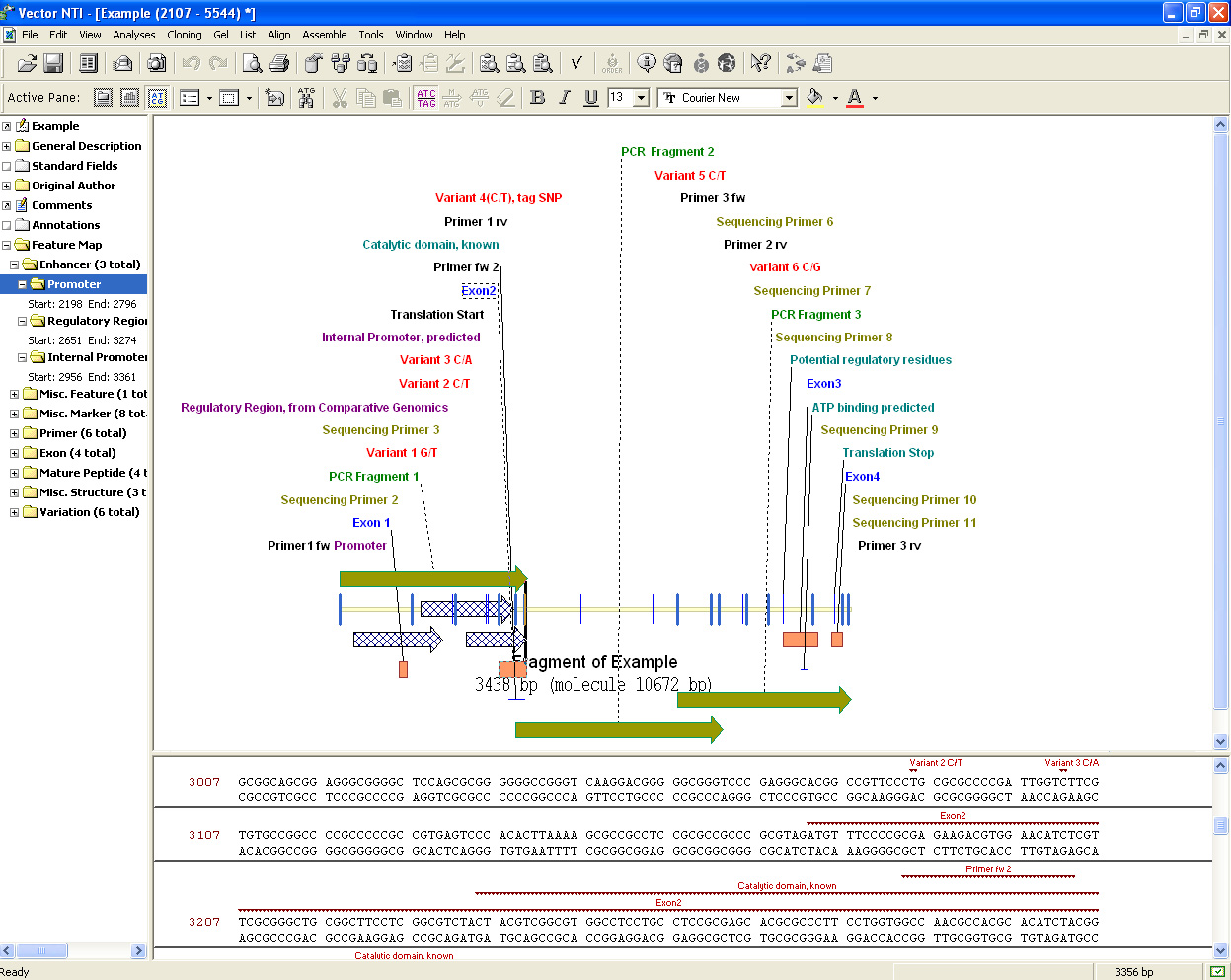

Supplement: Additional file 2 — This image shows a screenshot of a full Vector NTI data set prepared for Ecotilling, as described in figure legend 2. Figure 2 shows a section of this image. [file 1471-2164-9-510-S2.jpeg]
